# Optimized Zirconia 3D Printing Using Digital Light Processing with Continuous Film Supply and Recyclable Slurry System

**DOI:** 10.3390/ma14133446

**Published:** 2021-06-22

**Authors:** Waqas Ahmed Sarwar, Jin-Ho Kang, Hyung-In Yoon

**Affiliations:** 1 Department of Prosthodontics, School of Dentistry and Dental Research Institute, Seoul National University, Seoul 03080, Korea; engr.raowaqas@gmail.com; 2 Department of Prosthodontics, School of Dentistry, Chonnam National University, Gwangju 61186, Korea; jhk.bme1002@gmail.com

**Keywords:** zirconia, digital light processing, continuous film supply, recyclable slurry

## Abstract

Stereolithography (SL) can fabricate complex ceramic parts layer by layer using computer-aided design (CAD) models. The traditional SL system utilizes a vat filled with ceramic slurry with a high solid content, which for ceramics contributes to several limitations and operational difficulties, and further renders it nonrecyclable mainly due to the presence of printed residue and its high viscosity. In this study, we utilized a continuous film supply (CFS) system integrated with a tape-casting type digital light processing (DLP) printer to fabricate zirconia prototypes with a solid content of 45 volume percent (vol.%). Various printing and postprocessing parameters were studied for optimization, to achieve a relative density of 99.02% ± 0.08% with a microhardness of 12.59 ± 0.47 GPa. Slurry reusability was also demonstrated by printing with recycled slurry to produce consistent relative density values in the range of 98.86% ± 0.02% to 98.94% ± 0.03%. This method provides new opportunities for material recycling and the fabrication of dense complex ceramic products, reducing the consumption of the material.

## 1. Introduction

Zirconia ceramics provide unique properties in the field of biomedical applications. They demonstrate a low affinity to bacterial plaque, limit inflammatory infiltration, and provide good soft-tissue integration. However, compared to metals and polymers, high-strength ceramics such as zirconia are relatively difficult to process into intricate and complex structures via subtractive machining. Additive manufacturing (AM) fabricates three-dimensional (3D) objects based on computer-aided design (CAD) models by depositing printable materials layer by layer. The AM process can produce a variety of complex and intricate geometrical shapes simultaneously in a single workflow, without wasting machining tools and production time. With all these benefits, additively manufactured ceramic products are available using various technologies such as binder jetting, powder bed fusion, material extrusion, and vat polymerization [1,2,3]. Stereolithography (SL) or digital light processing (DLP) projector-based vat polymerization has especially high production accuracy and an excellent surface finish [4,5,6,7,8].

In vat-polymerization-based AM processes using ceramics, a high solid loading is required to fabricate fully dense ceramic objects, causing the aggregation of ceramic particles in the slurry and resulting in a high viscosity [9,10,11]. High viscosity produces challenges in maintaining accurate layer thickness and proper degassing. Although viscosity can be reduced by the use of diluents or temperature control, this is limited, as diluents can have adverse effects on the final density and produce high sintering shrinkage [12,13,14,15,16,17]. In addition, the recoating of the ceramic layer material during the printing recoating requires slurry of a low viscosity [18,19,20,21]. The homogenous temperature control of the whole vat area may help control the viscosity, though this is a challenging process [22].

Another major issue in vat polymerization with ceramics is light scattering by ceramic particles, resulting in the unwanted overgrowth of features that not only affects cure depth but also causes unexpected curing in bilateral directions [15,23,24,25,26,27]. Furthermore, the green printed state of ceramic material is fragile and lacking in the strength required to absorb the stresses involved in printing, making it susceptible to breakage during printing [28]. The presence of detached cured layers or partially failed samples in the vat poses hindrances for further printing, making the remaining slurry potentially non-reusable. Overall, vat polymerization faces many challenges in the slurry dispensing system, which cannot accommodate a higher viscosity without wasting slurry and processing time [29]. As an alternative method to vat polymerization, tape-casting-based additive manufacturing [22,24,28] can be utilized to build up ceramic materials layer by layer. It uses a casting head that acts as both a slurry reservoir and a material dispensing system. It is usually comprised of a single doctor blade that shears the slurry into a thin film onto a tape passing below the blades, providing a continuous film with uniform thickness [28]. It can also accommodate relatively higher-viscosity ceramics [28].

The purpose of this study was to produce high-density zirconia prototypes using a tape-casting type DLP printer with a continuous film supply by the optimization of processing parameters, and to evaluate the recycling efficiency of the system. The zirconia ceramic slurry used in this AM was characterized and processed for the complete printing workflow. The viscosity, film thickness, slurry recycling efficiency, thermogravimetry, shrinkage rate, density, and microhardness were assessed to evaluate the performance of the printing system.

## 2. Materials and Methods

### 2.1. Printer Design and Processing

**Figure 1:**
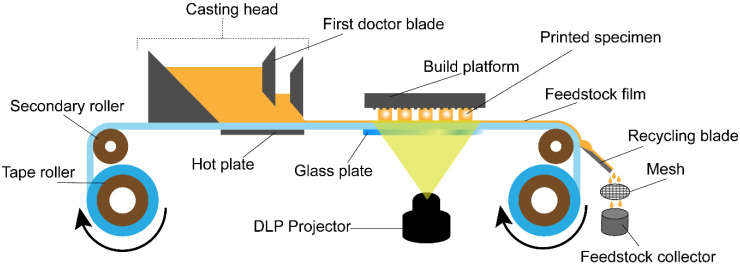
Schematic of the continuous film supply system with digital light processing during the three-dimensional printing process.

A modified version of a Onestage 6500 (Illuminaid Inc., Seoul, Korea) printer with a DLP projector was utilized for this research. The printer provides a continuous film of slurry to be printed in layers and is referred to as the “continuous film supply (CFS) system” throughout this study. The schematic design for this printing machine is shown in Figure 1. It mainly consists of a transparent hydrophobic tape conveyor coated with silicone material that moves from one roller to the other, passing through a casting head with dual doctor blades, a build platform, and a recycling blade. The film recoating thickness is controlled by physically controlling the double doctor blades and rolling speed. The projector installed in the DLP has an LED light source with a maximum irradiance of 5.19 mW/cm^2^ at a peak wavelength of 405 nm.

The system worked in sequences running successively, one after another. The first sequence initiated the rollers to move the tape for a set distance at a controlled speed, such that the slurry-recoated film arrived just under the build platform. The second sequence moved the build stage down to a set distance at a controlled speed, and decelerated close to its destination on the glass plate. The third sequence controlled the curing times for each designated set of layers of the whole process. The fourth sequence moved the platform up and the glass plate down, and finally, the manufacturing process repeated from the first sequence again. After hardening the selective area of the layer on the platform, the unused slurry was left on the tape. The unused slurry was peeled off from the tape using the recycling blade, where slurry flowed downward into the recycled-slurry collector after passing through a mesh of 100 µm to remove any agglomerations produced during printing, such as detached layers or damaged samples.

### 2.2. Slurry Preparation

A solvent-free photocurable ceramic suspension was formulated, containing high-purity ceramic powder dispersed in an organic resin composed of two multifunctional acrylate monomers, two photoinitiators, and some processing additives. A 3 mol % yttria-stabilized zirconia powder (TZ-3YS-E, Tosoh Corp, Tokyo, Japan) with an average particle size of 90 nm was used for this slurry. The zirconia ceramic powder was coated with a dispersant by ball milling in ethanol for 5 h and then dried in a vacuum oven at 30 °C. A nonvolatile hydroxy-functional ester-based dispersant was chosen for the zirconia ceramic suspension, based on the surface chemistry of the ceramic powder and resin. For this research, a ceramic suspension with a solid loading of 45 vol.% was prepared to balance the required density and printable viscosity. A suspension with a solid loading of 44.65 vol.% was chosen for the recycling experiment to ease the filtration and recycling process. The prepared ceramic suspension (slurry) was further homogenized before printing with a planetary centrifugal mixer (Thinky, ARE-310, Tokyo, Japan). The degassing process was then conducted in a −0.1 MPa vacuum for 5 min. The homogenization and degassing processes were also carried out for the recycled slurry.

### 2.3. Specimen 3D Printing

**Figure 2:**
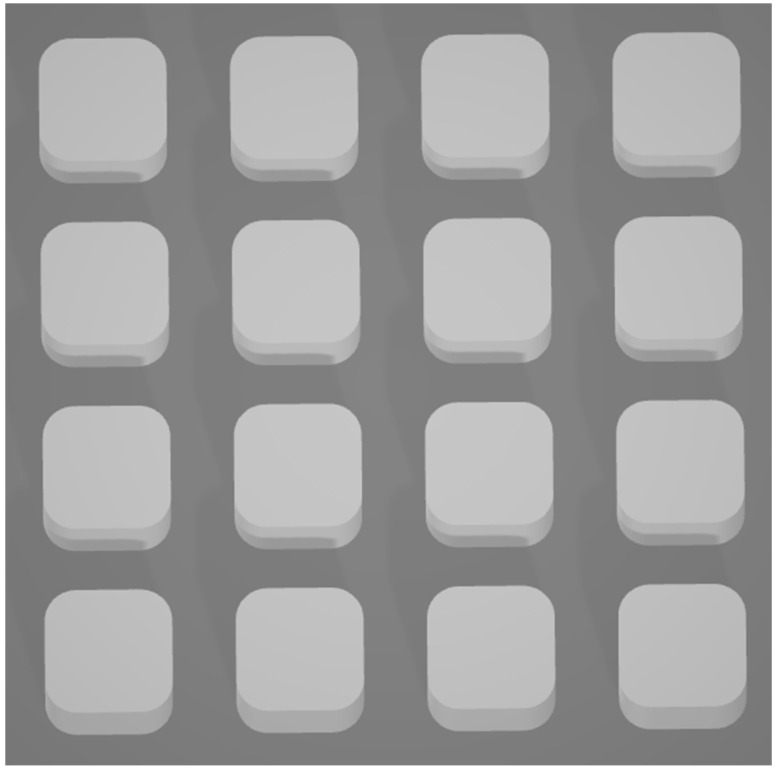
A standard tessellation language file image for the standard specimens to be printed.

The 3D printing of each specimen was performed according to the sequence mentioned above. The layer thickness was kept at 50 µm for this research. An exposure energy of 20.76 mJ/cm^2^ was applied to print each layer except for the first five layers, to which 62.28 mJ/cm^2^ was applied [30,31]. Other parameters used are mentioned in Table 1. The wait time mentioned in the table refers to the time the build platform spent on the slurry film just before and after curing, without changing its position. A standard tessellation language (STL) file format was used as a CAD model (Figure 2) with specimen dimensions of 7 × 7 × 4 mm^3^ scaled linearly in all dimensions, considering compensation for the overgrowth percentage and sintering shrinkage.

### 2.4. Postprocessing

**Figure 3:**
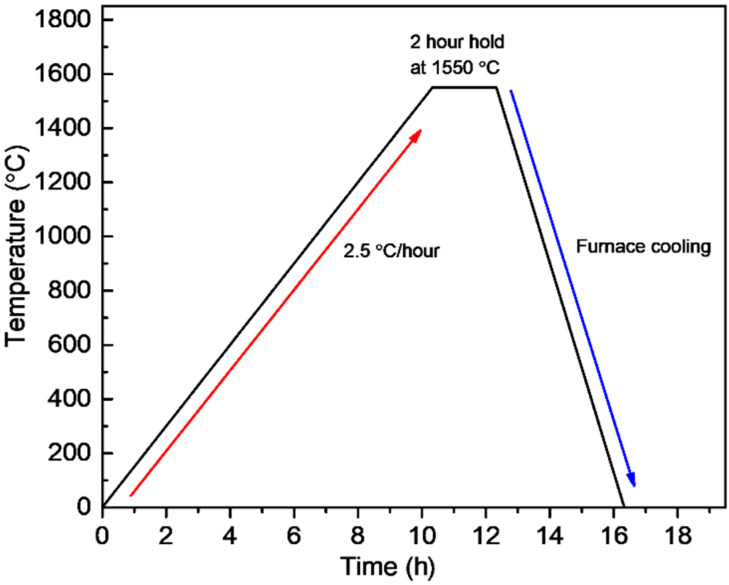
Sintering schedule followed by an atmosphere post debinding process in air.

All the printed specimens were cleaned using isopropanol through a handheld atomizing spray. Debinding was performed using an atmosphere type 1 furnace (PT-17EF022, PyroTech, Gyeonggi-do, Suwon si, Korea) in an argon atmosphere with a heating rate of 10 °C/h until 600 °C, with a dwell time of 2 h. A stairway approach to 600 °C was used with several holding temperatures, following derivative thermogravimetry analysis data. The sintering process was conducted in air at 1550 °C for 2 h with a heating rate of 2.5 °C/h, shown in Figure 3, using a Super Kanthal furnace (AJ-SKB6, Ajeon, Gyeonggi-do, Suwon si, Korea). The weight percent values of the solid loading of the specimens printed using the original ceramic slurry and the recycled slurry were measured from the weight difference between the specimens after debinding and sintering.

### 2.5. Viscosity Analysis

The viscosity of 2 mL of the ceramic slurry after degassing was measured at 30, 40, 50, and 60 °C using a rotational rheometer (TA Instruments, ARES-G2, New Castle, DE, USA).

### 2.6. Film Thickness Analysis of Slurry

The film thickness was measured using a micrometer (293-240-30, Mitutoyo, Kawasaki, Japan) with an accuracy of ±1 µm. The whole film above the projector was hardened and an average value was taken after taking measurements across the width. Measurement was repeated 5 times to calculate standard deviation and maintain a consistent slurry amount behind the blade.

### 2.7. Thermogravimetric Analysis

Simultaneous thermogravimetric–derivative thermogravimetry analysis (TG-DTG) was conducted using an analyzer device (SDT-Q600, TA Instrument, New Castle, DE, USA) on a printed cubic specimen with a size of 3 × 3 × 3 mm^3^, in argon atmosphere, and with a 1 °C/min temperature gradient from room temperature to 600 °C.

### 2.8. Density Analysis

The density of the printed specimen was characterized via the Archimedean principle using 10 replicates and an analytical balance (Adam Equipment, SAB 125i, Oxford, CT, USA) with readability of 10 µg. For recycled slurry, five replicates were used due to the limited amount of recycled slurry.

### 2.9. Microhardness Testing

The microhardness test value was measured by determining the Vickers hardness (Shimadzu, HMV-2, Kyoto, Japan) on mirror-polished cross-sections of the specimens, according to ASTM C1327-15 [32]. A loading force of 9.81 N was applied for 10 s, and the diagonals were measured through a ×40 objective lens. A total of 10 specimens were tested with at least 5 indents each, and the average value for those indents was considered as the final hardness value of each specimen.

### 2.10. Microstructure Analysis

The microstructure analysis was performed through a field-emission scanning electron microscope (AURIGA, Carl Zeiss, Oberkochen, Germany) on platinum-coated specimens with a coating thickness of 5 nm. The analysis was carried out on the fine-polished cross-sections of both as-printed specimens and sintered specimens after thermal etching at 1450 °C for 40 min. The grain size was measured for 5 sintered samples, printed in separate batches, by the linear intercept method with a conversion factor of 1.56 [33].

## 3. Results and Discussion

### 3.1. Viscosity Analysis

**Figure 4:**
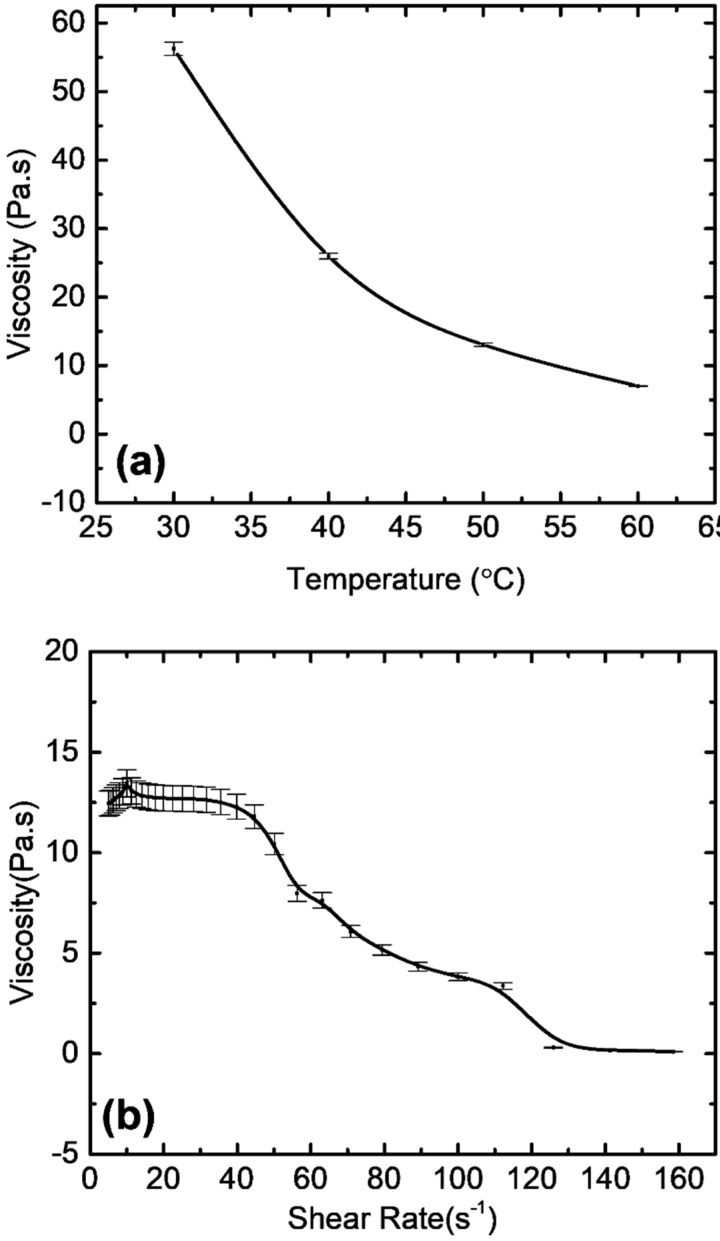
(**a**) Change in viscosity with the temperature at a 10 s^−1^ shear rate; (**b**) effect of shear rate on the viscosity of zirconia slurry at 50 °C.

Slurry with a 45 vol.% solid loading of zirconia coated with dispersant was tested at four different temperatures; however, it was found that the slurry started to thermally cure at temperatures higher than 60 °C. At around 60 °C, the slurry started to show signs of thermal curing and formed agglomerates which started to collect in the recycling mesh. The viscosity seemed to decrease exponentially with rising temperatures (Figure 4a). Viscosity decreased by a factor of four from 57 Pa·s at 30 °C to 13 Pa·s at 50 °C. The effect of the dispersant also played a role in this decrease in viscosity at higher temperatures, as the dispersant is most effective at temperatures higher than 30 °C. A working temperature of 50 °C was chosen, considering the lowest available viscosity without the possibility of the thermal curing of the slurry. The doctor blade requires a suitable viscosity range to make a homogenous film. To control and increase the printable viscosity range, temperature control and a proper dispersant of an optimized amount are essential [22,34]. The casting head, which acts as a slurry reservoir, has a hot plate below it to control temperature. Furthermore, a diluent can also be used to drastically reduce the viscosity; however, to avoid higher sintering shrinkage and to retain sufficient viscosity for the doctor blade, a diluent was not used.

At 50 °C, the viscosity was also tested with varying shear rates (Figure 4b). The viscosity decreased from 12.26 × 103 mPa·s at 3 s^−1^ to 96.95 mPa·s at 160 s^−1^. The flow curve was consistent with non-Newtonian flow behavior and showed pseudoplastic behavior. Considering that the tape velocity under the casting head was 8 mm/s, the viscosity was found to be in a suitable range for the doctor blades to sufficiently coat the slurry film at a uniform thickness.

### 3.2. Film Thickness of Slurry

**Figure 5:**
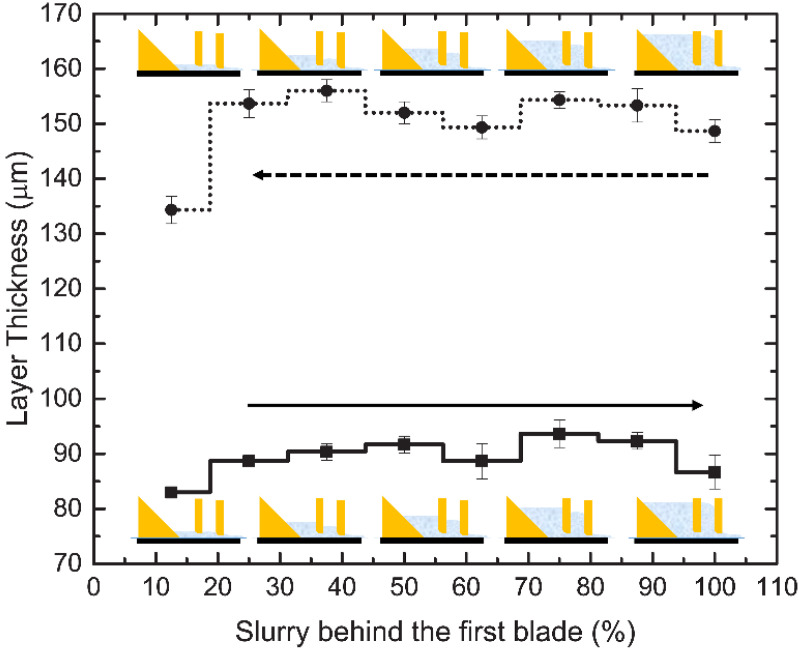
Film thickness measurement on the tape versus slurry level behind the first doctor blade. First with ascending level (**solid line**); second with descending level (**dotted line**).

Figure 5 shows the effect of the amount of slurry in the casting head behind the blades on the coated film thickness. It shows two trends, with the first trend (dotted) starting with 100% of the slurry reservoir filled with slurry and the second trend (solid) starting from 10% slurry. The dotted line depicts the natural process of recoating during printing with the slurry amount reducing as printing continues. The solid line depicts a trend when, at any point during printing, the reservoir was filled again with fresh or recycled slurry. Considering the dotted line, the film thickness stayed consistent with a mean difference of 5 ± 1 µm from slurry amounts of 100% (149 ± 2 µm) to 25% (154 ± 2 µm). For the solid line, the mean film thickness was between 86 ± 3 µm and 90 ± 3 µm, when the slurry amount varied from 20% to 100%, respectively. If a partial area was observed in the case of refill, the mean difference in film thickness was 4 ± 2 µm.

Film thickness depends on the viscosity, hydrodynamic pressure, tape speed, width of the blades, and density of the slurry [24,35,36]. Viscosity plays an important role and can change the flow pattern of slurry in diverse ways; however, with a consistent viscosity, the flow pattern changes with both Newtonian and non-Newtonian fluids only slightly [37]. In this study, the CFS utilized a dual-blade casting head, which in cases of variation in the slurry amount behind the first blade causes a minor change in film thickness if a sufficient tape velocity is maintained [38,39]. However, in the case of a non-steady state, the effect of slurry addition for the completion of the printing process (recycling) on the film thickness is also important. This addition of slurry, at any time, causes a sudden change in hydrodynamic pressure behind the blades, which can eventually affect film thickness. The effect of refilling the casting head was minute changes in film thickness unless it fell below a certain slurry height, which, in our case, was determined to be less than 25% (10 mm) of the total capacity. Layer thickness was controlled by the position of the build platform before curing. The build platform maintained its height for the programmed layer thickness. Recoated film was always fabricated 50–80 µm higher than the programmed layer thickness for compensation, which was then pushed out by compression by the build plate, to cure the exact layer thickness. Considering this alternative film recoating system of the doctor blades, the accurate layer thickness could be maintained in highly viscous slurry conditions, even when the slurry was below 10% capacity of the casting head, by the use of compensation. The excess slurry lost on the film was recovered by recycling.

### 3.3. Thermogravimetric Analysis

**Figure 6:**
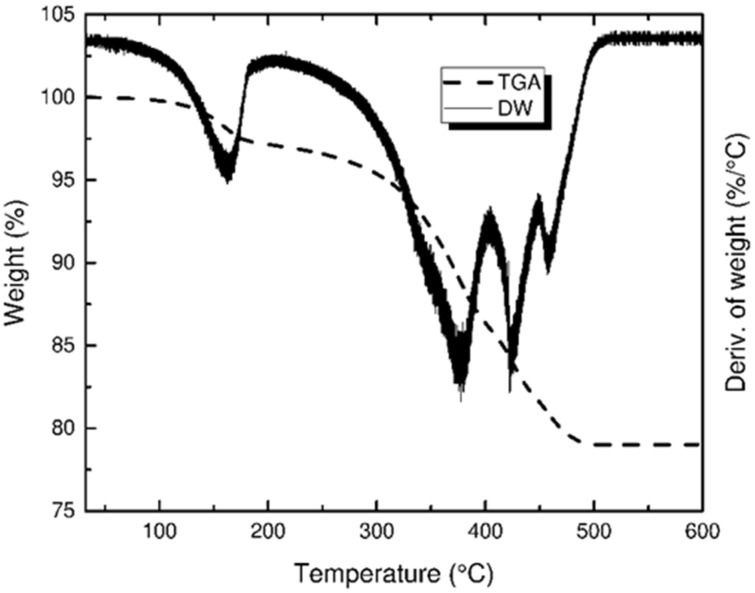
Thermogravimetric analysis representing both weight change percent and the rate of change in weight vs. temperature in an argon atmosphere.

Postprocessing after cleaning and drying started with binder burnout, which was carried out separately from sintering to check the effectiveness of the binder burnout and diagnose the initiation of possible thermal cracks. The derivative of weight (DW) curve shown in Figure 6 exhibits a sequence of peaks that indicate the presence of many phase changes during thermal degradation. Even though there are three distinctive peaks between 300 and 500 °C, their lower portions overlap with each other, which could, in some cases, hide smaller peaks in the overlapped regions. This makes the debinding process in this temperature zone heat sensitive, and it requires a comparatively lower heating rate. Considering the overlapping peaks in the DW curve, a heating rate of 10 °C/h in argon was utilized to eliminate thermal cracks, as an air atmosphere was found to cause thermal cracks after debinding, even at a slower heating rate of 5 °C/h.

Oxidative degradation produced mainly gaseous products that, under hindered diffusion, can create microcracks and blisters due to the pressure buildup of gas inside the sample. In the case of thermal degradation, polymer chains break down by chain scission with byproducts including carbon black, carbon monoxide, ammonia aliphatic amines, ketones, nitriles, and hydrogen cyanide [40]. Thermal degradation involves the removal of functional groups one after the other to leave behind only carbon black. Thermal degradation is a much slower process, and can be considered more sensitive as compared to oxidative degradation when crack formation during post-processes is compared. To avoid thermal stresses and cracks in sintered products, debinding was performed in an argon environment.

### 3.4. Densification

After cleaning, drying was performed at room temperature and no observable drying shrinkage was detected. In this study, 45 vol.% was the optimum solid loading in the argon atmosphere to achieve a relative densification of 99.02% ± 0.08% (5.99 ± 0.01 g/cm^3^) for zirconia. Linear shrinkage is shown in Table 2, which indicates homogenous sintering with shape retention and a lack of deformation during post-processing. Uniform sintering shrinkage in all dimensions is essential for any application, especially to produce an accurate final product. The shrinkage rate of 20–25% was within the normal limits for traditional CAD/CAM pre-sintered zirconia blocks [41,42].

### 3.5. Slurry Recycling Efficiency

All the residual slurry remaining on the tape was recycled after filtration and could be recycled again and used to refill the casting head after degassing for continuous printing. The characterization of samples printed using recycled slurry is given in Table 3. The results showed that there was a change in solid loading of 0.12 weight percent for the first recycling instance, and 0.26 weight percent for the second recycling instance. Changes in solid loading for recycled slurry can be explained by considering scattering agglomeration, which is the curing of slurry present near the exposed light area by light scattered by ceramic powder [24,43,44]. However, such a minute change in weight percent in a high-solid-load slurry was negligible. This was evident in the relative density of samples prepared only by using recycled slurry unmixed with fresh slurry, as there was only a slight change in density, making printing possible even after two instances of recycling. In practice, recycled slurry is mixed with the fresh slurry already present in the casting head, which can reduce solid loading changes further.

### 3.6. Microhardness Test

**Figure 7:**
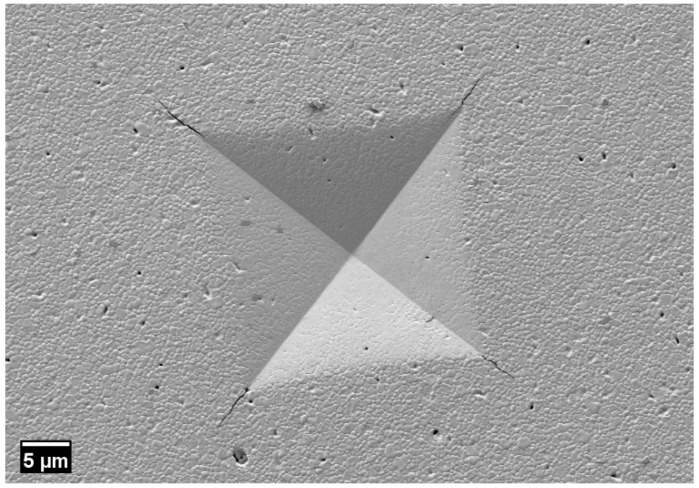
Indent at 1000× magnification, for reference.

Sintered specimens showed an average microhardness of 12.59 ± 0.47 GPa. As observed from Figure 7, the shape of the indent was symmetrical, the cracks produced at the corners of the square pyramid were linear, and it was well within the range of acceptable indentation by ASTM. Only indents producing Palmqvist cracks were measured for diagonals, and unsymmetrical indents were ignored during the measurement. The indentation seemed to include microporosity, as seen inside the pyramid; however, the porosity was not large enough to deform the indentation. A microhardness of over 12 GPa at 45 vol.% solid loading was well within the reported value produced by the additive manufacturing and conventional subtractive machining of zirconia plates [45,46].

### 3.7. Microstructure Analysis

**Figure 8:**
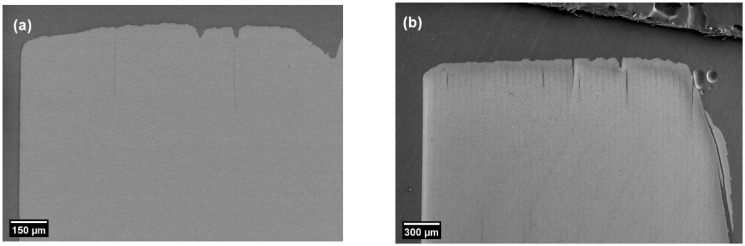
(**a**) Cross-section of sintered specimen at 200× (back-scattered electron mode) to observe any layer boundaries; (**b**) cross-section of as-printed specimen at 100× to observe distinctive layer boundaries.

**Figure 9:**
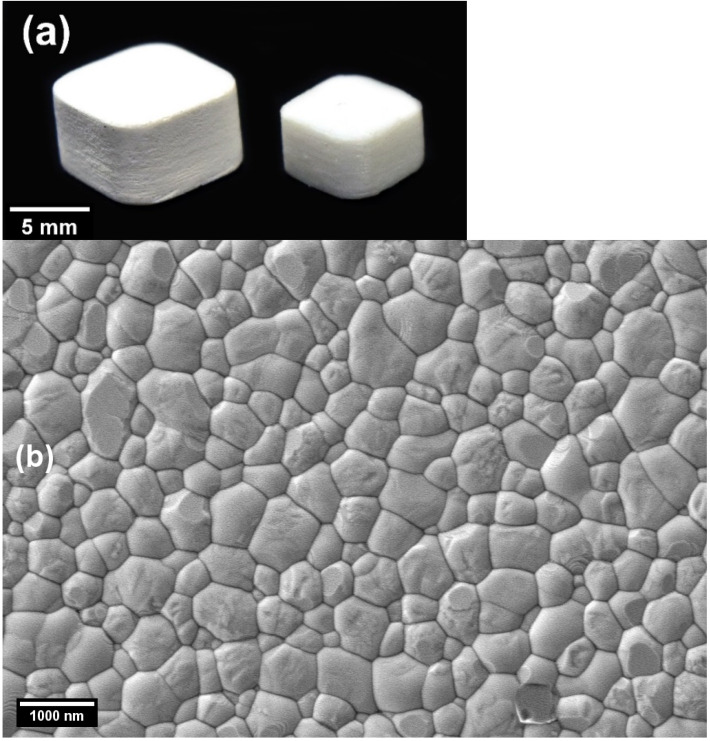
(**a**) As-printed and sintered samples for reference; (**b**) microstructure of the polished cross-section of 45 vol.% solid loaded zirconia at 30,000×.

The sintered cross-section did not show any signs of boundaries between layers, which were visible before debinding, as seen in Figure 8. This disappearance of layer boundaries after sintering is not common among traditional vat-polymerization printed parts. The disappearance of layer boundaries, after sintering and attaining a relative density of more than 99% at 45 vol.%, showed that tape casting produced compact as-printed objects. The DLP with CFS system showed promise in producing dense structures. The as-printed specimen is shown in Figure 9a as an example of printability. Furthermore, at 30,000× magnification the microstructure showed a mean grain size of 644 ± 20 nm and was densely packed, as evident in Figure 9b. However, further refinement of the grain size could be achieved by increasing solid loading and decreasing the sintering temperature.

## 4. Conclusions

Based on this study, the tape casting DLP printing with CFS is a capable alternative to vat polymerization to print dense zirconia prototypes using high-viscosity slurry. An appropriate dispersant and working temperature were necessary to achieve printable viscosity. Consistency of film thickness on the tape, throughout the whole printing process, was imperative for the DLP with CFS. Dual doctor blades were found to produce a film thickness well above the overall set layer thickness. Using the DLP with CFS, a 45 vol.% solid loaded 3Y-TZP slurry was used to fabricate ceramic products of a homogenous microstructure, with a relative density of 99.02% ± 0.08% and a mean grain size of 644 ± 20 nm after postprocessing. The microhardness value of the ceramic product was 12.59 ± 0.47 GPa. Furthermore, the DLP with CFS can effectively recycle and reuse slurry, providing a consistent density for at least two instances of recycling, making it a competitive alternative to conventional processing techniques for the fabrication of dense ceramic products.

## Figures and Tables

**Table 1:** Several parameters used for printing.

Parameter	Value
Wait time before curing	12 s
Wait time after curing	5 s
Tape speed	8 mm/s
Build platform speed (z-direction)	1 mm/s
Height of first blade	1 mm
Height of second blade	0.2 mm

**Table 2:** Sintering shrinkage in all directions.

*X*-Direction (%)	*Y*-Direction (%)	*Z*-Direction (%)
22.59 ± 0.19	22.48 ± 0.15	22.56 ± 0.11

**Table 3:** Change in properties with recycling.

Properties	First Use	Recycled Once	Recycled Twice	Recycled Thrice
Weight percent (%)	81.32 ± 0.03	81.44 ± 0.04	81.58 ± 0.06	81.61 ± 0.02
Relative Density (%)	98.89 ± 0.03	98.86 ± 0.02	98.94 ± 0.03	98.91 ± 0.03

## Data Availability

The data presented in this study are available on request from the corresponding author.
